# A glimpse of diabetic nephropathy

**Published:** 2016-01-24

**Authors:** Farhad Khoshjou

**Affiliations:** Urology and Nephrology Research Center, Hamadan University of Medical Sciences, Hamadan, Iran

**Keywords:** Diabetic nephropathy, Kimmelstiel–Wilson nodules, Glomerulosclerosis

**Implication for health policy/practice/research/medical education:**

Prevalence of diabetes has been increasing in last decades. Diabetic nephropathy (DN), on the other hand, is turn out to be the most frequent cause of end stage renal disease (ESRD) worldwide. Mechanisms as well as the management of diabetic nephropathy, have been studied deeply. Here a glimpse of them is presented.

Worldwide, the prevalence of diabetes was estimated to 382 million in 2013; and is projected to reach 592 million by 2035. This represents 8%–10% of the global population (1). Diabetic nephropathy (DN), on the other hand, is becoming the most frequent cause of end-stage renal disease (ESRD) in most countries. It is characterized by pathological magnitude of urine albumin excretion and loss of glomerular filtration rate (GFR). Structural changes includes mesangial expansion, thickening of the glomerular basement membrane (GBM) and characteristically, nodular glomerulosclerosis (Kimmelstiel–Wilson nodules) (2). Pathogenesis of DN, not surprisingly, has been studied from different aspects.

It consists of, but not limited to: (*a*) Hemodynamic: Renin-angiotensin system (RAS) role, has been well known in the pathogenesis of DN (3). (*b*) Metabolic: Oxidative stress and generation of reactive oxygen species (ROS) damage DNA and proteins (4). Besides activation of the polyol pathway, by converting excess glucose to sorbitol, contributes to aforementioned phenomenon (5). Moreover, formation of advanced glycation end-products (AGE) by non-enzymatic binding of glucose to proteins lead to alteration of proteins structure and function (6). (*c*) Growth factors/cytokines: Activation of TGF-β and its downstream cytokine, connective tissue growth factor (CTGF), induces extracellular matrix formation and fibrosis. (*d*) Cellular organelles: Mitochondrion, the power plant of cell, is directly involved in and injured by oxidative stress. Deficient mitochondrial oxidative phosphorylation and decreased mitofusin-2 expression in patients with type 2 diabetes and obesity have been found (7). The role of endoplasmic reticulum stress in diabetic kidney disease has been confirmed, as well (8). (*e*) Trace elements: Zinc, and chromium have been shown to significantly decrease in blood samples of DN cases compared to healthy subjects (9).

## Management

### Angiotensin-converting-enzyme inhibitor (ACE) inhibitors and angiotensin receptor blockers (ARBs)

While earlier studies of combination ACE inhibitor and ARB reported dominance of grouping therapy for lowering albuminuria and blood pressure against either alone (10). Recent studies have confirmed the combination treatment was associated with higher incidences of acute renal failure and hyperkalemia (11).

### Aldosterone antagonist

Bearing in mind that aldosterone endorses fibrosis, inflammation, and generation of ROS, Spironolactone, as expected, reduces proteinuria (12).

### Hypoglycemic agents

Good glycemic control is effective in reducing diabetic microvascular complications. Besides, DPP-4 inhibitors (gliptins) have shown anti-inflammatory and anti-apoptotic properties in diabetic kidney disease. For instance, in type 2 diabetics, sitagliptin treatment for 6 months diminishes albuminuria independent of HbA1c (13).

### Statins

Experimentally, statins have been shown to lessen AGE-mediated ROS activation, tubular apoptosis and suppress RAS activation (14).

### Vitamin D

PRONEDI trial of type 2 diabetics showed that, vitamin D deficiency is a risk factor for ESRD in DN cases (15).

### Novel agents

A couple of novel agents including glycosaminoglycans (sulodexide) are under investigation (16).

## Author’s contribution

FK is the single author of the manuscript.

## Conflicts of interest

The author declared no competing interests.

## Ethical considerations

Ethical issues (including plagiarism, data fabrication, double publication) have been completely observed by the author.

## Funding/Support

None.
